# Whole-Genome Assemblies of 56 *Burkholderia* Species

**DOI:** 10.1128/genomeA.01106-14

**Published:** 2014-11-20

**Authors:** H. E. Daligault, K. W. Davenport, T. D. Minogue, K. A. Bishop-Lilly, S. M. Broomall, D. C. Bruce, P. S. Chain, S. R. Coyne, K. G. Frey, H. S. Gibbons, J. Jaissle, G. I. Koroleva, J. T. Ladner, C.-C. Lo, C. Munk, G. F. Palacios, C. L. Redden, C. N. Rosenzweig, M. B. Scholz, S. L. Johnson

**Affiliations:** a Los Alamos National Laboratory (LANL), Los Alamos, New Mexico, USA; b Diagnostic Systems Division (DSD), United States Army Medical Research Institute of Infectious Diseases (USAMRIID), Fort Detrick, Maryland, USA; c Naval Medical Research Center (NMRC)-Frederick, Fort Detrick, Maryland, USA; d Henry M. Jackson Foundation, Bethesda, Maryland, USA; e United States Army Edgewood Chemical Biological Center (ECBC), Aberdeen Proving Ground, Aberdeen, Maryland, USA; f Center for Genome Sciences (CGS), USAMRIID, Fort Detrick, Maryland, USA

## Abstract

*Burkholderia* is a genus of betaproteobacteria that includes three notable human pathogens: *B. cepacia*, *B. pseudomallei*, and *B. mallei*. While *B. pseudomallei* and *B. mallei* are considered potential biowarfare agents, *B. cepacia* infections are largely limited to cystic fibrosis patients. Here, we present 56 *Burkholderia* genomes from 8 distinct species.

## GENOME ANNOUNCEMENT

*Burkholderia* is a diverse genus of Gram-negative aerobic bacilli that was previously considered part of the genus *Pseudomonas* (1). Of the 82 recognized *Burkholderia* species, three are notable human pathogens: *B. pseudomallei*, *B. mallei*, and *B. cepacia* (an opportunistic lung infection pathogen in cystic fibrosis patients).

*B. pseudomallei*, the causative agent of melioidosis, is an environmental bacterium commonly found in southeastern Asia and northern Australia, where it causes multiple annual outbreaks. *B. mallei* is an obligate pathogen that primarily causes disease in horses, mules, and donkeys (called farcy) but is also the causative agent of glanders in humans, which can be either an acute or chronic infection (1). Both *B. pseudomallei* and *B. mallei* are on the CDC category B list due to their low infectious dose and high mortality rates, even with antibiotic therapy (2). While no evidence exists for the weaponization of *B. pseudomallei*, *B. mallei* has been developed as a weapon (2), and with their high transmission and mortality rates, both are considered potential biowarfare agents (3–5).

We sequenced the genomes of two *Burkholderia cenocepacia*, four *B. cepacia*, one *Burkholderia gladioli*, eight *B. mallei*, two *Burkholderia multivorans*, one *Burkholderia oklahomensis*, 34 *B. pseudomallei*, two *Burkholderia thailandendsis*, one *Burkholderia xenovorans*, and one strain not identified to species level. These additions will increase the publicly available scaffolded and completed genomes by 11 to 100% for each species (46% over all species).

High-quality genomic DNA was extracted from 100-ml bacterial cultures of purified isolates for each strain using the Qiagen Genomic tip-500, per the manufacturer’s recommendations, with one minor variation. For biosafety level 3 (BSL3) *Burkholderia* organisms, all cultures were lysed overnight to ensure the sterility of the resulting extracted material. If sterility was not achieved, the nucleic acid was passed through a 0.45-µM filter and rechecked for viable organisms before removal from the BSL3 suite.

The sequence data for each draft genome include at least two data types: Illumina (6), 454 (7), and for some, PacBio (8) technologies. The draft genome coverages for each data type are included in the NCBI submission records; however, the Illumina (either unpaired or short-insert) coverages ranged from 114- to 1,067-fold, and the 454-based long-insert (insert size range, 5.2 to 12.7 kb) coverages were generally <50-fold. The combined draft data had genome coverages between 134- and 1,186-fold. The 454 and Illumina data were assembled together using Newbler and Velvet. All draft data were assembled together with AllPaths (9), and if the PacBio data were available and at ≥100× coverage, they were assembled using HGAP (10). The consensus sequences from all assemblers were computationally shredded and assembled with a subset of read pairs from the long-insert library using Phrap (11, 12). The resulting assemblies were manually and computationally improved using Consed (13) and in-house scripts.

The annotations were completed using the Ergatis workflow manager (14) and in-house scripts. The genomes are available in NCBI, and the raw data can be provided upon request. In-depth comparative analyses of these and other genomes will be published in subsequent reports.

### Nucleotide sequence accession numbers.

The genome accession numbers to public databases are listed in Table 1.

**TABLE 1:** Accession numbers and basic assembly statistics for each assembled *Burkholderia* genome

Strain	Alternate ID^*a*^	Source	Accession no. (no. of contigs)	Size (bp)/G+C (%)	No. of CDS^*b*^
*Burkholderia cenocepacia*					
DDS 22E-1	BHS	Australia, aerosol, 2005	CP007782-CP007784	8,045,250/67.0	7,088
DWS 37E-2	BHT	Australia, soil, 2007	CP007779-CP007781	6,612,421/66.5	5,775
*Burkholderia cepacia*					
ATCC 25416	BGF	United States, plant, 1948	CP007745-CP007748	8,567,011/66.6	7,739
DDS 7H-2	BHR	Australia, aerosol, 2005	CP007785-CP007787	8,147,114/67.1	7,337
DWS 16B-4	BHX	Australia	JPGE00000000 (4)	8,112,163/67.1	7,289
DWS 37UF10B-2	BHW	Australia, soil, 2007	JPGD00000000 (6)	7,182,032/67.2	6,400
*Burkholderia gladioli*					
ATCC 25417	ICPB PM 2	Plant	JPGG00000000 (18)	9,311,425/67.4	8,044
*Burkholderia mallei*					
092700E	NCTC 10247	Turkey, 1960	CP007801 and CP007802	5,827,656/68.5	5,001
ATCC 23344	China 7, BMF	Burma, human, 1944	CP008704 and CP008705	5,625,292/68.5	4,883
BMK	ATCC 15310	Hungary, horse, 1961	CP008731 and CP008732	5,872,022/68.5	5,069
BMQ	106	India, horse, 1932	CP008722 and CP008723	5,630,231/68.5	4,892
BMY	6	Turkey, human, 1950	CP008710 and CP008711	5,647,769/68.5	4,872
BMZ	ATCC 10399	China, horse, 1956	JPNX00000000 (3)	5,856,639/68.5	5,031
FMH	BGL	Burma, human, 1944	CP009147 to CP009148	5,835,541/68.5	5,026
SR092700I	BMP	NA^*c*^	JNLV00000000 (246)	5,675,037/68.5	5,236
*Burkholderia multivorans*					
DDS 15A-1	BHQ	Australia, aerosol, 2005	CP008729 and CP008730	7,281,867/66.6	6,529
DWS 42B-1	BHV	Thailand, soil, 2007	JNLW00000000 (6)	6,505,001/67.3	5,773
*Burkholderia oklahomensis*					
BDU	E0147	United States, human	CP008726 and CP008727	7,313,673/66.9	6,312
*Burkholderia pseudomallei*					
BDD	DSTO T18	Australia, human, 1996	JNOW00000000 (80)	7,361,146/68.0	6,206
BDE	DSTO T21	Australia, human, 1997	JPNW00000000 (118)	7,253,846/68.1	6,144
BDI	DSTO T27	Australia, human, 1998	JPNU00000000 (76)	7,268,791/68.1	6,106
BDM	DSTO T30	Australia, human, 1998	JPNV00000000 (102)	7,495,075/67.9	6,411
BDT	DSTO T43	Australia, human, 1999	JOTS00000000 (53)	7,358,678/67.9	6,143
BDZ	DSTO T43	Australia, human, 1996	JPNO00000000 (357)	7,296,307/68.0	6,519
BEB	DSTO T82	Australia, human, 2000	JPNP00000000 (142)	7,310,901/68.1	6,367
BEC	DSTO T87	Australia, human, 2000	JOTX00000000 (248)	7,533,026/68.0	6,797
BED	DSTO T2	Australia, human, 1996	JPNQ00000000 (254)	7,244,575/68.0	6,418
BEF	DSTO T14	Australia, human, 1996	JPNR00000000 (232)	7,297,941/68.0	6,473
BEG	DSTO T17	Australia, human, 1996	JOTY00000000 (245)	7,445,118/67.9	6,604
BEH	DSTO T106	Australia, human, 2001	JOTZ00000000 (187)	7,362,104/67.9	6,413
BEJ	PHLS 112	Thailand	JPNS00000000 (4)	7,198,519/68.2	5,884
BEK	9	Pakistan	CP008754 and CP008755	7,228,737/68.1	5,978
BEM	Pasteur 52237	Vietnam	JPNT00000000 (9)	7,358,404/68.0	6,090
BEO	1106a	Thailand, human, 1993	CP008758 to CP008759	7,086,433/68.3	5,758
BES	DSTO T75	Australia, human, 2000	JPHA00000000 (305)	7,720,797/67.6	7,070
BEX	MSHR576A	Thailand	CP008777 and CP008778	7,266,604/68.0	5,944
BEZ	MSHR1655	NA	CP008779 and CP008780	7,027,950/68.0	5,798
BFB	MSHR346	NA	CP008763 and CP008764	7,354,216/67.9	6,044
BFD	DSTO T9	Australia, human, 1996	JOTT00000000 (67)	7,343,224/67.9	6,201
BGH	DSTO T88	Australia, human, 2000	JOTU00000000 (15)	7,506,190/67.9	6,269
BGQ	576a	Thailand	JOTV00000000 (79)	7,245,828/67.9	6,128
BGR	1026b	NA	CP008834 and CP008835	7,231,385/68.2	5,960
BGS	1106b	Thailand	JOTW00000000 (52)	7,077,890/68.2	5,853
BGV	DSTO T6	Australia, human, 1996	JPHB00000000 (23)	7,451,876/67.9	6,204
BSR	406e	NA	CP009127 and CP009128	7,272,702/68.1	5,941
HBPUB10134a	BHN	Thailand, human, 2010	CP008911 and CP008912	7,218,403/68.1	5,858
HBPUB10303a	BHO	Thailand, human, 2011	CP008893 and CP008894	7,178,176/68.2	5,834
Mahidol-1106a	BGI	Thailand	CP008781 and CP008782	7,085,397/68.3	5,748
MSHR305	BDP	Human, 1994	CP009209 and CP009210	7,442,161/67.9	6,144
MSHR5848	BHL	Australia, human, 2011	CP008909 and CP008910	7,290,434/68.1	5,989
MSHR5855	BHK	Australia, human, 2011	CP008783 and CP008784	7,297,804/68.0	6,001
MSHR5858	BHM	Australia, human, 2011	CP008891 and CP008892	7,070,528/68.3	5,781
*Burkholderia thailandensis*					
BDK	2003015869	United States, human	CP008914 and CP008915	6,728,580/67.7	5,709
BTY	E264	Thailand, soil, 1994	CP008785 and CP008786	6,722,099/67.6	5,655
*Burkholderia xenovorans*					
BXA	LB400, BXA	United States, soil, 1985	CP008760 and CP008762	9,702,951/62.6	8,684
*Burkholderia* sp.					
BGJ	1710a	Thailand	JOUA00000000 (9)	5,472,690/67.9	5,983
BGK	1710b	Thailand	CP008916 and CP008917	7,304,000/68.0	5,962

a ID, identification.

b CDS, coding sequences.

c NA, not available.
